# Supplementary material for: Cross-sectional seroprevalence surveys of SARS-CoV-2 antibodies in children in Germany, June 2020 to May 2021
Source: Nat Commun. 2022 Jun 6;13:3128. doi: 10.1038/s41467-022-30482-6 (PMC9170697; doi:10.1038/s41467-022-30482-6)
Supplement: Supplementary file 1 — Supplementary Information [file 41467_2022_30482_MOESM1_ESM.pdf]

SUPPLEMENTARY INFORMATION

Cross-sectional seroprevalence surveys of SARS-CoV-2 antibodies in children in Germany, June 2020 to May 2021

Sorg *et al.*

Corresponding Author: Rüdiger von Kries (Mail: Ruediger.kries@med.uni-muenchen.de)

Batches used of the SARS-CoV-2 ELISA 1
Supplementary Methods 2
SARS-CoV-2-S1 Enzyme-Linked Immunosorbent Assay (ELISA) 2
Plaque Reduction Neutralisation Tests 2
Qualification of serology in children 2
Supplementary Tables 4
Supplementary Figures 6
English version of the two-page study-specific questionnaire 10
Supplementary References 12

Batches used of the SARS-CoV-2 ELISA

First application date	Date of expiry	Batch number
28/4/2020	22/10/2020	E200423AF
3/6/2020	23/10/2020	E200422AK
25/6/2020	27/10/2020	E200428BI
26/6/2020	02/11/2020	E200503AD
9/7/2020	18/11/2020	E200519CF
14/7/2020	02/11/2020	E200503AD
15/7/2020	28/10/2020	E200429AG
20/7/2020	17/10/2020	E200428BX
30/7/2020	18/11/2020	E200519CF
25/11/2020	18/10/2021	E201019RQ
11/12/2020	12/11/2021	E201113BP
22/12/2020	08/12/2021	E201209BZ
3/3/2021	12/1/2022	E210113BH
29/3/2021	17/1/2022	E210118BQ
20/4/2021	03/3/2022	E210304BV
29/4/2021	11/4/2022	E210412AN
7/6/2021	21/4/2022	E210422AW
17/6/2021	17/5/2022	E210518BP
8/7/2021	20/5/2022	E210521BZ
19/8/2021	20/5/2022	E210521CA
12/10/2021	24/8/2022	E210825BR

Supplementary Methods

SARS-CoV-2-S1 Enzyme-Linked Immunosorbent Assay (ELISA)

A commercially available SARS-CoV-2 S1 ELISA (EUROIMMUN Medizinische Diagnostika AG; Lübeck, Germany) was used according to the manufacturer's instructions. Serum samples were analysed at a 1:101 dilution using the fully automated EUROLabWorkstation ELISA platform. A serum was considered reactive at an optical density (OD) ratio ≥ 1.1. OD ratio is the quotient of the OD in a sample and the OD of a calibrator. The calibrator is a pooled European adult pre-pandemic serum included as a serum reference in every run. OD Ratio can be used as a semi-quantitative measure for the concentration and affinity of serum antibodies.

Plaque Reduction Neutralisation Tests

PRNTs were performed according to an in-house protocol, as described.[1] 24-well plates were seeded with Vero E6 cells (1.6 x10^5 cells/well) and incubated overnight. Sera were diluted in a log2 sequence using OptiPro and mixed 1 : 1 with 100 µL authentic virus (Munich isolate 984) containing 50 plaque forming units. The first dilution was 1 : 20. 100 µL of serum was added to each 24-well and incubated for 1 hour at 37°. Any supernatant was discarded, and cells were washed once with PBS and supplemented with 1.2% Avicel solution in DMEM. After 3 days at 37°C, the supernatants were removed, and the 24-well plates were fixed and inactivated using a 6% formaldehyde/PBS solution and stained with crystal violet. In duplicates, we assessed all serial serum dilution causing plaque reductions of 90% (PRNT-90) and 50% (PRNT-50). A PRNT-50 ≥ 1 : 20 was classified as positive for the presence of SARS-CoV-2 neutralising antibodies.

Qualification of serological testing in children

Most children, when infected with SARS-CoV-2, remain asymptomatic or mildly symptomatic, so estimating the prevalence of SARS-CoV-2 in children by testing symptomatic patients by RT-PCR imprecise.[2–4] However, in adults, most studies agree that the Immunoglobulin G (IgG) antibodies persist beyond the acute phase of infection.[5] Therefore, serosurveys can reveal prior infection on a population basis, as IgG-antibodies against parts of the SARS-CoV-2 spike protein persist over months in most people previously infected with SARS-CoV-2.[6–8]

After infection, children mount an antibody response against the SARS-CoV-2 S1 domain at a rate similar to that of adults.[9,10] Anti- SARS-CoV-2 S1 IgG was therefore chosen to test for prior SARS-CoV-2 infections in children.

The optimal serologic method of detecting antibodies against SARS-CoV-2 in children is unknown. We used a commercially available anti-SARS-CoV-2 assay based on the detection of IgG in serum by ELISA. The assay was developed and manufactured by Euroimmun AG (Lübeck, Germany) and was one of the first commercially available assays in Germany. The Euroimmun platform was shown to have a high correlation with the PRNT, regarded as the gold standard for the detection of antibodies.[11] In contrast to PRNT, ELISA systems are capable of analysing serum samples at high throughput required for a national serosurvey, as conducted here. Due to the time needed for seroconversion and waning of antibodies, the diagnostic sensitivity of the assay varies with time after infection and reaches 94.4% in adult sera collected >10 days after infection.

Previous studies assessed the diagnostic properties of the ELISA Euroimmun and verified the sensitivity of the test and the detectability of IgG antibodies over time.[11,12] Therefore, we confined these analyses to this test. The prevalence estimate depends not only on the applied test, but equally importantly on the chosen cut-off value.

Therefore, we took two approaches to qualify ELISA results in our study population: Using data on the presence of neutralising antibodies and the reported infection status obtained though the questionnaire, we evaluated the manufacturer-recommended ELISA threshold and evaluated whether alternative thresholds better predict putative infections in children by Receiver Operating Characteristic (ROC) analysis.

(1) In populations where seroprevalence is low, lack of test specificity results in a high rate of false positives. We confirmed the detection of SARS-CoV-2 specific antibodies using an authentic virus PRNT. Here, we tested 143 ELISA reactive sera with OD ratios ≥ 1.1 (Median OD ratio: 2.07; range: 1.1 - 11.54) and 109 ELISA negative sera (Median OD ratio: 0.87; Range: 0.27 - 1.09) for the presence of antibodies by plaque reduction neutralisation test. Overall, 55 / 252 sera had a PRNT titer ≥ 1 : 20. Among ELISA negative sera, PRNT-50 was ≥ 1 : 20 in 3 / 109 (2.75%). All three PRNT50 positive sera were within the borderline (0.8 - 1.1) OD ratio range of the applied ELISA system. In 52 / 143 (36.4%) ELISA positive children, the PRNT titer was ≥ 1 : 20. In line with that, the median OD ratio in all 55 sera with a PRNT-50 ≥ 1 : 20 was significantly higher than in sera with a PRNT-50 < 1 : 20, 3.51 vs. 1.05 respectively (p = 0.0001, Fig. 5A and 5B).

We performed ROC-curve analysis to determine a threshold OD ratio that separates sera by PRNT titer with the highest sensitivity and specificity. ROC analysis resulted in an optimal OD ratio threshold to predict neutralising antibodies of 2.04 (95% CI 1.69, 2.36). The maximal Youden's J statistic was 0.7. ROC-curve AUC was 0.91. At the threshold of 2.04, sensitivity was 85.2% (95% CI 72.9, 93.4) and specificity was 84.9% (95% CI 77.6, 90.5) for the detection of a titer ≥ 1 : 20 by PRNT (Fig. 5C).

(2) SARS-CoV-2 infection status was reported in the questionnaire. A total of 4073 / 10,358 patients were previously tested for SARS-CoV-2 infection and 167 / 4073 patients reported a SARS-CoV-2 infection had been previously diagnosed (Fig. 5D). We used ROC-curve analysis to determine a threshold OD ratio that best separates previously infected as reported in the questionnaire from those that did not report a previous SARS-CoV-2 infection. Infection status per questionnaire was best predicted at a threshold of ≥ 0.61 (95% CI 0.35, 0.85), with Youden's J statistic of 0.71. At this threshold, sensitivity was 77.3% (95% CI 70.1, 83.4) and specificity was 93.5% (95% CI 92.6, 94.2) for detecting a patient with a questionnaire-reported prior infection (Fig. 5C and 5D).

(3) At the manufacturer-recommended threshold of 1.1, sensitivity and specificity to predict a PRNT-50 ≥ 1 : 20 were 94.4% (95% CI 84.6, 98.8) and 41.6% (95% CI 32.9, 50.8), respectively. The manufacturer's threshold predicted infection status per questionnaire with a sensitivity of 71.3% (95% CI 63.8%, 78.0%) and specificity of 95.6 (95% CI 94.9, 96.2).

Sensitivity and specificity with reference to a preceding SARS-CoV-2 infection based on recalled previous positive test results in the questionnaire refer to 'a best available arbitrary standard' and not to a 'gold standard', which must be taken into account when interpreting the values.

The AUC values of the ROC curves were similar for the prediction of neutralising PNTR titres and preceding positive SARS-CoV-2 test. The sensitivity and specificity varied depending on cut-off values accounting for different absolute estimates for seroprevalence. We used the manufacturer-recommended cut-off value to maximise specificity and to enable better comparison with other studies.

All three thresholds were used to calculate point estimates of seroprevalence. As higher thresholds are less sensitive, the seroprevalence depends on the applied cut-off values. For example, at the end of our study period, a minimum estimate of seroprevalence is 7.5% (95% CI 5.1, 10.0) using the 2.04 cut-off. The maximal estimate of seroprevalence is 13.1% (95% CI 10.0, 16.2) at the 0.61 cut-off.

The temporal trends of seroprevalence throughout the study period and the predicted probability of a positive serum test according to b-spline regression models (Fig. 6) were similar for all three tested thresholds, confirming the robustness of the applied ELISA test. Therefore, we assume that any cut-off within the range of OD ratios of 0.61 and 2.04 delivers an acceptable estimate of the seroprevalence trend. Despite similar trends, it must be acknowledged, as mentioned, that the total seroprevalence estimates at any point of the observation period would be substantially shifted depending on the chosen cut-off value. The higher the cut-off, the lower the seroprevalence estimates. The strength of this study design is to depict the temporal course and to suggest a reasonable range for the seroprevalence at a given point in time. We chose the manufacturer-recommended threshold at OD ratio 1.1 for our analysis, because it permits comparison with adult serosurveys using the same system.

Supplementary Tables

Supplementary Table 1: Comparison of characteristics of the study participants with and without previous test results

	Participants with previous test results N=4073			Participants without previous test results N=6050			
	N[*]		%[+]	N[*]		%[+]	p-value[#]
Sex	4068			6041			0.56
Male		2018	49.6		2961	49.0	
Female		2050	50.4		3080	51.0	
Age group	4073			6050			0.26
< 3 years		606	14.9		830	13.7	
3 to 11 years		1843	45.3		2777	45.9	
12 to 17 years		1624	39.9		2443	40.4	
Language spoken in the family	3925			5828			0.11
German		3526	89.8		5292	90.1	
Other Language		399	10.2		536	9.2	
Country of origin of the parents	3936			5861			0.14
Germany (both parents)		2384	60.6		3635	62.0	
Germany (one parent)		532	13.5		716	12.2	
Outside Germany (both parents)		1020	25.9		1510	25.8	
Reason for hospitalisation	3336			4171			<0.0001*
Planned treatment		983	29.4		711	17.1	
Routine check-up		1022	30.6		1881	45.1	
Referral for inpatient evaluation or parent/patient education		442	13.3		371	8.9	
Emergency		598	17.9		833	20.0	
Other		291	8.7		375	9.0	
Respiratory infection or pneumonia as current reason for hospitalisation	3904	132	3.4	5826	155	2.7	0.04
History of respiratory diseases since March 2020	3970	770	19.4	5926	645	10.9	<0.0001*
History of pneumonia since March 2020	4014	141	3.5	5950	83	1.4	<0.0001*
History of hospitalisation due to pneumonia since March 2020	3686	93	2.5	5496	45	0.8	<0.0001*
Past medical history (pre-existing conditions)	4013	2491	62.1	5947	3233	54.4	<0.0001*
Selected pre-existing conditions							
Asthma	3550	267	7.5	5292	357	6.8	0.16
Mucoviscidosis	3531	63	1.8	5264	104	2.0	0.52
Bronchopulmonary dysplasia (BPD)	3481	33	1.0	5233	47	0.9	0.81
Heart disease / heart defect	3559	206	5.8	5303	212	4.0	<0.0001*
Haematological / oncological disease	3525	241	6.8	5263	245	4.7	<0.0001*
Neurological / neuromuscular disease	3537	378	10.7	5244	326	6.2	<0.0001*
Congenital or acquired immunodeficiency	3460	94	2.7	5216	115	2.2	0.42
Autoimmune disease	3501	337	9.6	5282	616	11.7	0.003
Metabolic disease	2966	225	7.6	3721	296	8.0	0.58
Gastrointestinal disease	2946	322	10.9	3703	287	7.8	<0.0001*
Chronic renal disease	2956	148	5.0	3675	112	3.1	<0.0001*

[*] Difference to the absolute number of recruits is due to unanswered questions.
[+] Percentage not adding up to 100% is explained by rounding.
[#] Two-sided exact p-values for Pearson chi-square (p-values marked with * are <0.05 after Bonferroni correction) for the association to SARS-CoV-2 IgG antibodies.
[‡] questionnaire did not specify the applied test method.
Abbr: N - Number of participants with available information; SARS-CoV-2 - Severe Acute Respiratory Syndrome Coronavirus 2; IgG - immunoglobulin G

Supplementary Table 2: Crude two-month-average Severe Acute Respiratory Syndrome Coronavirus 2 seroprevalence as determined by an Enzyme-Linked Immunosorbent Assay and seroprevalence estimates standardised for migrant background (at least one parent with country of origin outside Germany), age group, and study site

	**Number of observed events/Total** (n=9922) Missing information on parents' country of origin n=436	**Crude SARS-CoV-2 seroprevalence** in % (95% CI)	**Standardised SARS-CoV-2 seroprevalence** for migrant background, age groups, and study sites in % (95% CI)
Apr/Jun 2020	55/2130	2.54 (1.87, 3.20)	3.07 (2.05, 4.09)
Aug/Sep 2020	29/1960	1.48 (0.95, 2.01)	1.74 (0.79, 2.69)
Oct/Nov 2020	41/1882	2.18 (1.52, 2.84)	1.87 (1.23, 2.52)
Dec 20/Jan 21	87/1494	5.82 (4.64, 7.01)	5.25 (3.64, 6.85)
Feb/Mar 2021	140/1502	9.32 (7.85, 10.79)	10.76 (7.59, 13.93)
Apr/May 2021	73/954	7.65 (5.96, 9.34)	7.56 (4.76, 10.36)

Abbr: SARS-CoV-2 - Severe Acute Respiratory Syndrome Coronavirus 2

Supplementary Figures

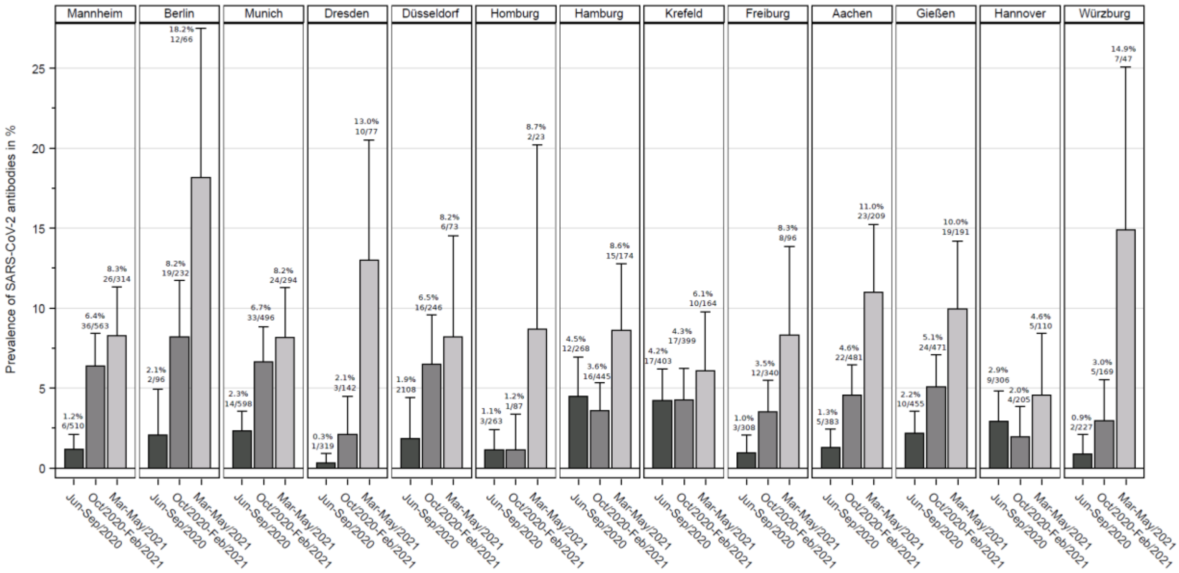

Supplementary Fig. 1: Prevalence of Severe Acute Respiratory Syndrome Coronavirus 2 (SARS-CoV-2) immunoglobulin G antibodies as determined by an enzyme-linked immunosorbent assay in blood samples, from in total 10,358 pediatric study participants, in the different stages of the COVID-19 pandemic in Germany stratified by study center. The height of the boxes displays the respective point estimates of the prevalences and the whiskers indicate the upper 95% confidence limit of these point estimates.

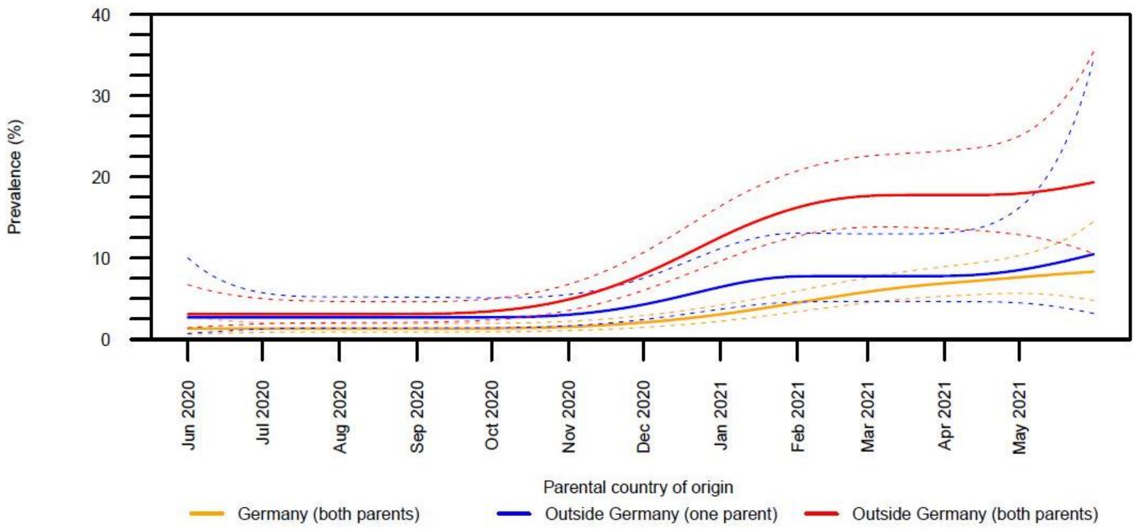

Supplementary Fig. 2: Predicted probability of Severe Acute Respiratory Syndrome Coronavirus 2 seropositivity according to b-spline regression models (with 95% confidence bands - dashed lines), as determined by an Enzyme-Linked Immunosorbent Assay in children from May 2020 to May 2021 stratified by migrant background.

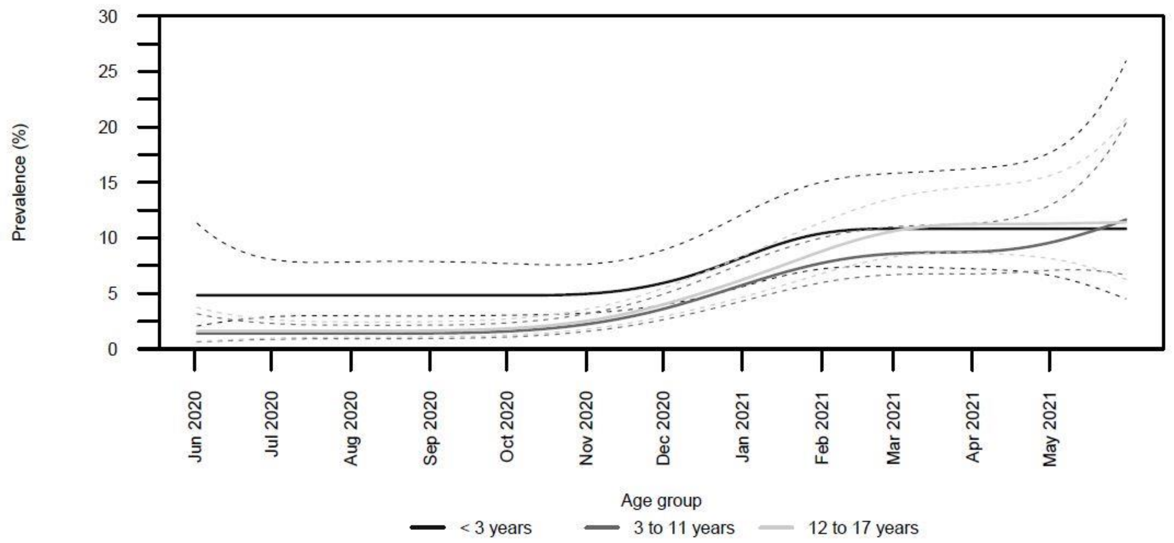

Supplementary Fig. 3: Predicted probability according to b-spline regression models (solid lines) with 95% confidence bands (dashed lines) of Severe Acute Respiratory Syndrome Coronavirus 2 immunoglobulin G antibodies as determined by an enzyme-linked immunosorbent assay, stratified by age group.

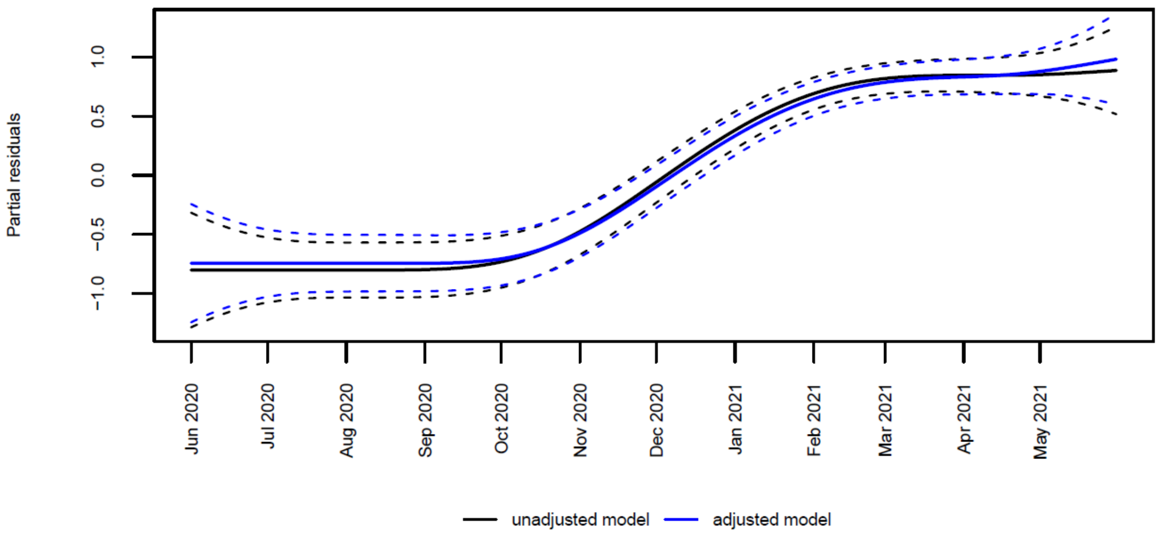

Supplementary Fig. 4: Partial residuals (with 95% confidence bands - dashed lines) of the logistic regression model for Severe Acute Respiratory Syndrome Coronavirus 2 seropositivity with time as non-linear b-spline function (black curve) and the partial residuals of this logistic regression model adjusted (blue curve) for migrant background (reference Germany as country of origin of both parents) and age group (reference 12 to 17 years old).

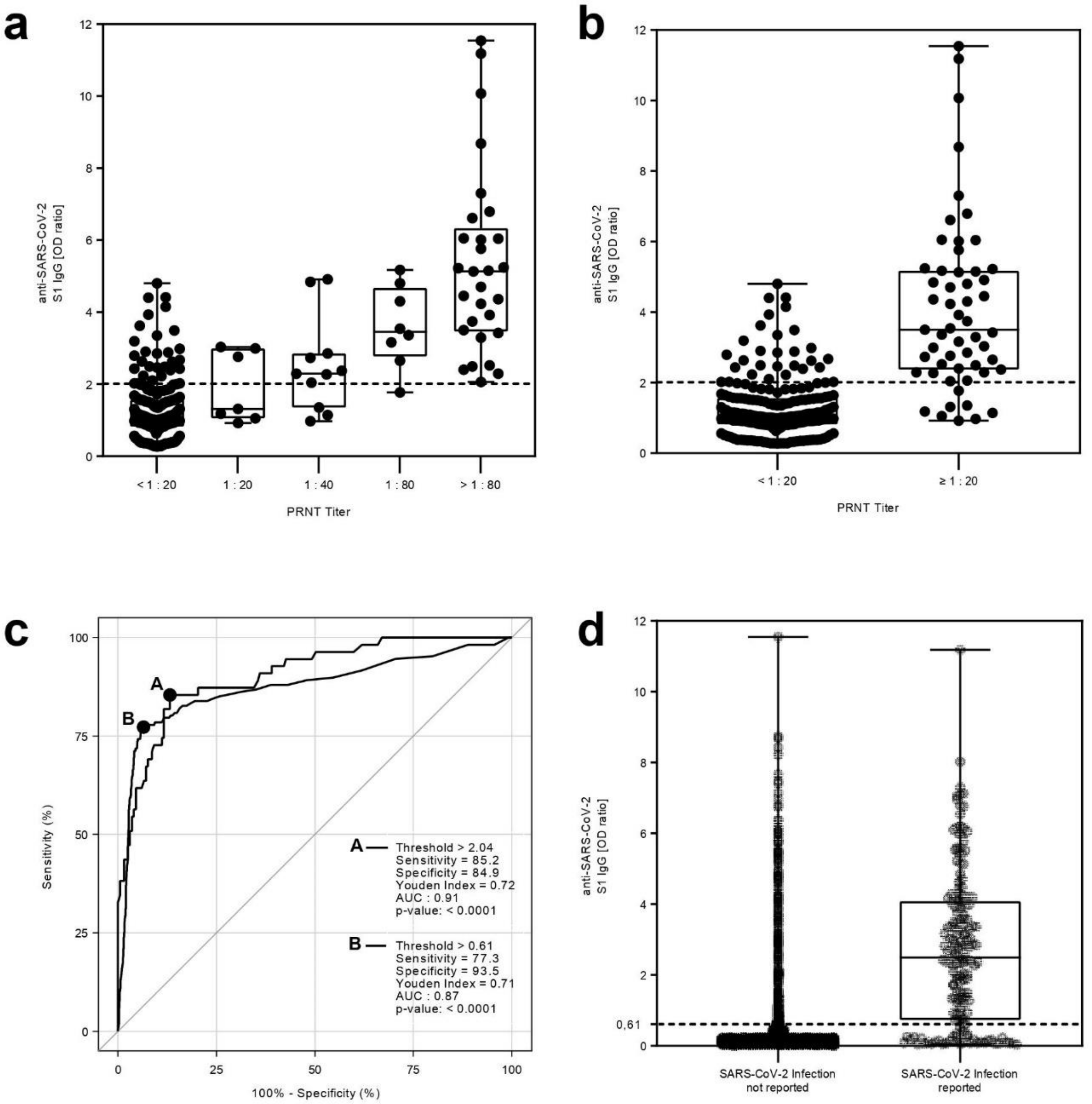

Supplementary Fig. 5: Qualification of serological testing in children. (A and B) Plaque Reduction Neutralisation Tests in a subset of 179 sera samples. (PRNT)-50 Titre increases with an increasing median of anti-Severe Acute Respiratory Syndrome Coronavirus 2 optical density (OD) ratio. An OD ratio of 2.04 (dashed line) best separates sera with and without neutralising antibodies. (C) Using Receiver Operating Characteristic (ROC) analysis, threshold A was determined to have the highest sensitivity and specificity for predicting neutralising antibodies. Threshold B was determined to have the highest sensitivity and specificity to predict participant infection status per questionnaire. Information of a preceding test result of SARS-CoV-2 infection was available for 4073 participants, of whom 167 reported preceding positive test results (D). The middle lines in the boxplots in (A), (B) and (D) indicates the median, the boxes respectively indicate the first and third quartile, the whiskers the 1.5 interquartile range (IQR). The dots display the individual values. Two-Sided p-values in (C) are based on Mann-Whitney (Wilcoxon) Test and indicate statistical significance of the area under the curve (AUC, Null hypothesis is AUC=0.5).

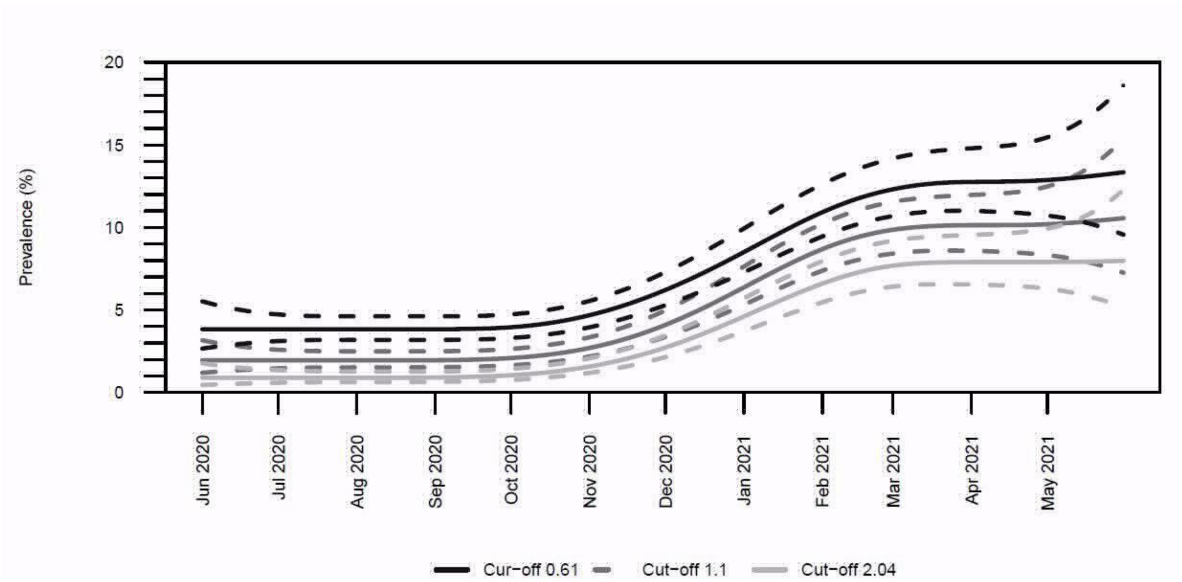

Supplementary Fig. 6: Predicted probability of Severe Acute Respiratory Syndrome Coronavirus 2 seropositivity according to b-spline regression models (with 95% confidence bands - dashed lines), as determined by an Enzyme-Linked Immunosorbent Assay in children from May 2020 to May 2021 with different cut-offs of ELISA optical density ratio.

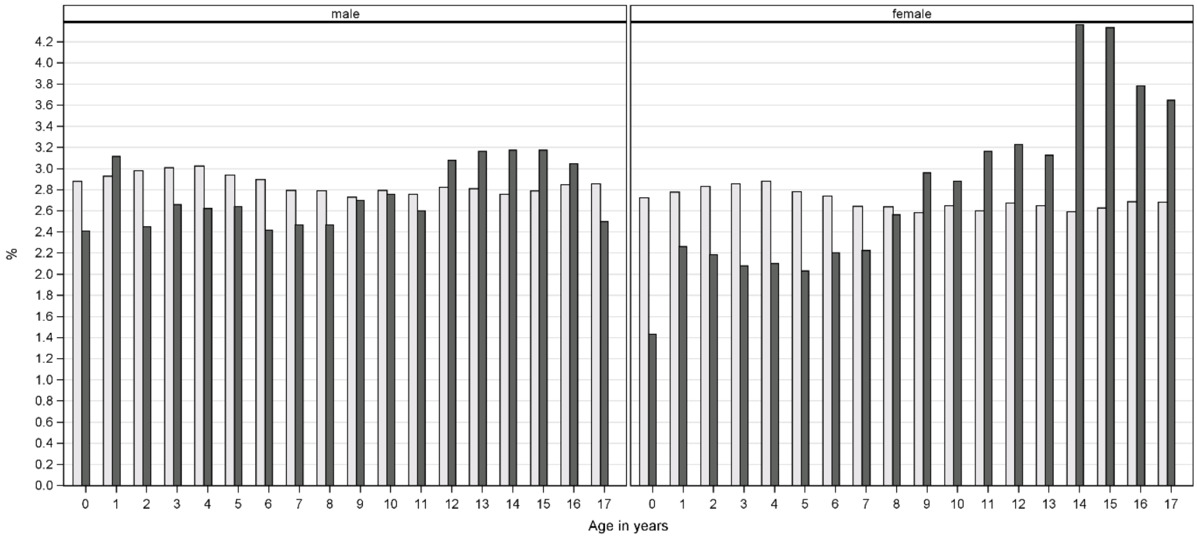

Supplementary Fig. 7: Age distribution of the German population and the study population, displayed by sex. In the years 2020 13,743,944 children aged 0 to 17 years lived in Germany, 7,063,395 male and 6,680,549 female children (light grey). The study population includes in total 10,338 children, 5110 male and 5228/ female (dark grey).

English version of the two-page study specific questionnaire

(D) Questionnaire	Field for barcode sticker

"Research on the spread of the new coronavirus in children and adolescents in Germany."

1. Date of answering the questionnaire

__ __ | __ __ | __ __ __ __
day month year

Please enter the date on which you complete the questionnaire/ the blood draw takes place.

2. Sex

☐ female ☐ male

3. Birthdates of the child

Month of birth _ _ (example: month of birth January = 0 1)

Year of birth _ _ _ _ (example: year of birth 2014 = 2 0 1 4)

4. Country of origin of the parents

Country of origin mother: ____________________ (please name)

Country of origin father: ____________________ (please name)

Language spoken in the family

☐ German ☐ other: ________________

5. Reason for consulting a doctor in hospital

What is the reason for the blood draw of your child?

☐ Planned treatment (surgery, MRI, Endoscopy etc.)
☐ Routine check-up (in the outpatient clinic)
☐ Referral for inpatient evaluation or parent/patient education
☐ Emergency
☐ Other reason, which one? ____________________

Is the reason for the current treatment of your child in the paediatric hospital a respiratory infection or pneumonia?

☐ yes ☐ no ☐ I do not know

6. Infectious diseases

How many respiratory infections⁺ with the symptoms of fever* and shortness of breath has your child gone through since 1st March 2020 until today?

☐ none ☐ one ☐ two
☐ three ☐ four ☐ five
☐ six or more ☐ I do not know

* fever=body temperature >38°C
⁺ A respiratory infection is a cold, bronchitis or flu

How often has pneumonia been diagnosed in your child since 1st March 2020 until today? (please name only if the diagnosis was made by a physician)

☐ never ☐ one ☐ twice
☐ more than twice ☐ I do not know

Did any of these pneumonia diagnoses require hospital treatment since 1st March 2020?

☐ yes ☐ no ☐ I do not know

If yes, how often? ____ times

Has your child had fever for more than 48 hours since the beginning of March 2020 for no obvious reason?

☐ yes ☐ no ☐ I do not know

If yes, have the following symptoms also been present?

- Skin rash (exanthema) or skin/mucosal inflammation ☐ yes ☐ no ☐ I do not know
- Conjunctivitis ☐ yes ☐ no ☐ I do not know
- Low blood pressure (hypotension) ☐ yes ☐ no ☐ I do not know
- Circulation failure (shock) ☐ yes ☐ no ☐ I do not know
- Features of myocardial dysfunction or heart inflammations/ abnormalities ☐ yes ☐ no ☐ I do not know
- Blood clotting disorder (coagulopathy) ☐ yes ☐ no ☐ I do not know
- Acute gastrointestinal problems (diarrhoea, vomiting, abdominal pain or suspected appendicitis) ☐ yes ☐ no ☐ I do not know

Has your child been hospitalised in a clinic because of this febrile illness?

☐ yes ☐ no ☐ I do not know

7. New Coronavirus Covid-19

Has your child been tested for SARS-CoV-2 (new coronavirus) the past?

☐ yes ☐ no ☐ I do not know

If yes, what was the result of this test?

☐ positive ☐ negative ☐ I do not know

Have one or more close family members (living in the same household) been suspected of being infected with SARS-CoV-2 (new coronavirus) in the past?

☐ yes ☐ no ☐ I do not know

Was SARS-CoV-2 (new coronavirus) detected in one of these close family members in the past (test positive)?

☐ no ☐ yes, in one close family member
☐ yes, in more than one close family members
☐ I do not know

Next on the back ↷

Study Centres:

Medical study direction:
Prof. Dr Horst Schroten
University Children's Hospital Mannheim
Theodor-Kutzer-Ufer 1-3 |
68167 Mannheim

Datacenter and statistics:
Prof. Dr. Ruediger von Kries (retired)
LMU Munich - Institute for Social Pediatrics and Youth Medicine
Haydnstrasse 5 | 80336 Munich

Study coordination / Study lab:
Prof. Dr. Christian Drosten
Charité University Medicine Berlin
Institute for Virology
Charitéplatz 1 | 10117 Berlin

8. Underlying disease

Does your child have any pre-existing conditions?

☐ yes ☐ no ☐ I do not know

Has your child ever had the following diseases?
(please tick as appropriate, multiple answers possible)

Asthma	☐ yes ☐ no ☐ I do not know
Mucoviscidosis	☐ yes ☐ no ☐ I do not know
Bronchopulmonary dysplasia (BPD)	☐ yes ☐ no ☐ I do not know
Other lung disease?	Which one? ___________________________
Heart disease / heart defect	☐ yes ☐ no ☐ I do not know
Haematological / oncological disease	☐ yes ☐ no ☐ I do not know
Neurological / neuromuscular disease	
Congenital or acquired immunodeficiency	☐ yes ☐ no ☐ I do not know
Autoimmune disease	☐ yes ☐ no ☐ I do not know
Metabolic disease	☐ yes ☐ no ☐ I do not know
Gastrointestinal disease	☐ yes ☐ no ☐ I do not know
Chronic renal disease	☐ yes ☐ no ☐ I do not know
Any other disease?	☐ yes ☐ no ☐ I do not know<br>Which one? ___________________________

How often was your child hospitalised because of one of these underlying diseases in the past year?

☐ never ☐ once ☐ twice
☐ more than twice ☐ I do not remember

Thanks a lot for participating in this study!

Study Centres:

Medical study direction:
Prof. Dr Horst Schroten
University Children's Hospital Mannheim
Theodor-Kutzer-Ufer 1-3 |
68167 Mannheim

Datacenter and statistics:
Prof. Dr. Ruediger von Kries (retired)
LMU Munich - Institute for Social Pediatrics and Youth Medicine
Haydnstrasse 5 | 80336 Munich

Study coordination / Study lab:
Prof. Dr. Christian Drosten
Charité University Medicine Berlin
Institute for Virology
Charitéplatz 1 | 10117 Berlin

Supplementary References

1. Wölfel, R. *et al.* Virological assessment of hospitalised patients with COVID-2019. *Nature* **581,** 465–469; 10.1038/s41586-020-2196-x (2020).
2. Tönshoff, B. *et al.* Prevalence of SARS-CoV-2 Infection in Children and Their Parents in Southwest Germany. *JAMA Pediatr* **175,** 586–593; 10.1001/jamapediatrics.2021.0001 (2021).
3. Parri, N., Lenge, M. & Buonsenso, D. Children with Covid-19 in Paediatric Emergency Departments in Italy. *N Engl J Med* **383,** 187–190; 10.1056/NEJMc2007617 (2020).
4. Viner, R. M. *et al.* Susceptibility to SARS-CoV-2 Infection Among Children and Adolescents Compared With Adults: A Systematic Review and Meta-analysis. *JAMA Pediatr* **175,** 143–156; 10.1001/jamapediatrics.2020.4573 (2021).
5. Long, Q.-X. *et al.* Antibody responses to SARS-CoV-2 in patients with COVID-19. *Nature Medicine* **26,** 845–848; 10.1038/s41591-020-0897-1 (2020).
6. Stadlbauer, D. *et al.* Repeated cross-sectional sero-monitoring of SARS-CoV-2 in New York City. *Nature* **590,** 146–150; 10.1038/s41586-020-2912-6 (2021).
7. Chen, Y. *et al.* A comprehensive, longitudinal analysis of humoral responses specific to four recombinant antigens of SARS-CoV-2 in severe and non-severe COVID-19 patients. *PLoS pathogens* **16,** e1008796; 10.1371/journal.ppat.1008796 (2020).
8. Munitz, A. *et al.* Rapid seroconversion and persistent functional IgG antibodies in severe COVID-19 patients correlates with an IL-12p70 and IL-33 signature. *Sci Rep* **11,** 3461; 10.1038/s41598-021-83019-0 (2021).
9. Knabl, L. *et al.* High SARS-CoV-2 seroprevalence in children and adults in the Austrian ski resort of Ischgl. *Commun Med* **1**; 10.1038/s43856-021-00007-1 (2021).
10. Buonsenso, D. *et al.* Seroprevalence of anti-SARS-CoV-2 IgG antibodies in children with household exposure to adults with COVID-19: Preliminary findings. *Pediatr Pulmonol* **56,** 1374–1377; 10.1002/ppul.25280 (2021).
11. Jahrsdörfer, B. *et al.* Independent Side-by-Side Validation and Comparison of 4 Serological Platforms for SARS-CoV-2 Antibody Testing. *J Infect Dis* **223,** 796–801; 10.1093/infdis/jiaa656 (2021).
12. Robertson, L. J. *et al.* Evaluation of the IgG antibody response to SARS CoV-2 infection and performance of a lateral flow immunoassay: cross-sectional and longitudinal analysis over 11 months. *BMJ open* **11,** e048142; 10.1136/bmjopen-2020-048142 (2021).
