# Supplementary material for: Cross-sectional seroprevalence surveys of SARS-CoV-2 antibodies in children in Germany, June 2020 to May 2021
Source: Nat Commun. 2022 Jun 6;13:3128. doi: 10.1038/s41467-022-30482-6 (PMC9170697; doi:10.1038/s41467-022-30482-6)
Supplement: Supplementary file 3 — Reporting Summary [file 41467_2022_30482_MOESM3_ESM.pdf]

nature portfolio

Corresponding author(s): Rüdiger von Kries

Last updated by author(s): 25.03.2022

Reporting Summary

Nature Portfolio wishes to improve the reproducibility of the work that we publish. This form provides structure for consistency and transparency in reporting. For further information on Nature Portfolio policies, see our Editorial Policies and the Editorial Policy Checklist.

Statistics

For all statistical analyses, confirm that the following items are present in the figure legend, table legend, main text, or Methods section.

n/a	Confirmed	
☐	☒	The exact sample size (*n*) for each experimental group/condition, given as a discrete number and unit of measurement
☐	☒	A statement on whether measurements were taken from distinct samples or whether the same sample was measured repeatedly
☐	☒	The statistical test(s) used AND whether they are one- or two-sided<br>*Only common tests should be described solely by name; describe more complex techniques in the Methods section.*
☐	☒	A description of all covariates tested
☐	☒	A description of any assumptions or corrections, such as tests of normality and adjustment for multiple comparisons
☐	☒	A full description of the statistical parameters including central tendency (e.g. means) or other basic estimates (e.g. regression coefficient) AND variation (e.g. standard deviation) or associated estimates of uncertainty (e.g. confidence intervals)
☐	☒	For null hypothesis testing, the test statistic (e.g. *F*, *t*, *r*) with confidence intervals, effect sizes, degrees of freedom and *P* value noted<br>*Give P values as exact values whenever suitable.*
☒	☐	For Bayesian analysis, information on the choice of priors and Markov chain Monte Carlo settings
☒	☐	For hierarchical and complex designs, identification of the appropriate level for tests and full reporting of outcomes
☒	☐	Estimates of effect sizes (e.g. Cohen's *d*, Pearson's *r*), indicating how they were calculated

Our web collection on statistics for biologists contains articles on many of the points above.

Software and code

Policy information about availability of computer code

Data collection	No software was used for data collection (pen and pencil data collection and manual data transfer to Microsoft Access database)
Data analysis	Code availability: The code used to generate the results is available online under https://osf.io/am2ck/ (DOI 10.17605/OSF.IO/AM2CK).<br><br>Software used in analysis and to generate the figures:<br><br>SAS, version 9.4 (SAS Institute Inc., Cary, NC, USA); R 3.5.1 (R Foundation for Statistical Computing, Wien, Austria)<br>R 3.6.2. (R Foundation for Statistical Computing,Wien, Austria).<br>Python 3.8<br>Graphpad Prism

For manuscripts utilizing custom algorithms or software that are central to the research but not yet described in published literature, software must be made available to editors and reviewers. We strongly encourage code deposition in a community repository (e.g. GitHub). See the Nature Portfolio guidelines for submitting code & software for further information.

Data

Policy information about availability of data

All manuscripts must include a data availability statement. This statement should provide the following information, where applicable:

- Accession codes, unique identifiers, or web links for publicly available datasets
- A description of any restrictions on data availability
- For clinical datasets or third party data, please ensure that the statement adheres to our policy

Data supporting the findings of this study are available from the corresponding author, RvK, upon reasonable request.

Field-specific reporting

Please select the one below that is the best fit for your research. If you are not sure, read the appropriate sections before making your selection.

☐ Life sciences ☒ Behavioural & social sciences ☐ Ecological, evolutionary & environmental sciences

For a reference copy of the document with all sections, see nature.com/documents/nr-reporting-summary-flat.pdf

Behavioural & social sciences study design

All studies must disclose on these points even when the disclosure is negative.

Study description	Quantitaitve, hospital-based, multicenter, study including cross-sectional seroprevalence surveys
Research sample	In order to obtain nationwide data and to attain a large sample size of children (aged <18 years), the recruitemtn of the study participants took place in 14 pediatric hospitals distributed in different federal states of Germany. Spare blood from blood samples taken for other medical purposes was choosen to increase compliance and to avoid study related adverse effects for the children. Repeated participation was not possible. The reason for the blood sampling or the visit int the hospital was not relevant for study participation.
Sampling strategy	convenience Sampling; A 95% confidence interval and the assumption of an infinite population were used to estimate the required sample sizes. It was assumed that the infection rate will change over the course of the study. In the first month of the survey, an infection rate of 5% is expected. It was assumed that after approximately 1 year, a significant proportion of children and adolescents will be infected with the SARS-CoV-2 (Covid-19) virus. The number of cases needed depends on the actual prevalence of infected persons and the desired precision of the estimate. The calculation was done with Epitools (https://epitools.ausvet.com.au/oneproportion). Sample size is calculated using the formula: n = (Z2 x P x (1 - P))/e2
Data collection	Data were collected by blood samples and questionnaires. The questionnaire was filled out by parents/guardians or patients, when appropriate for their age. The parents agreed to provide blood taken during a blood sampling for other clinical procedures of their children for study purposes. A unique identifier was used to link blood samples and the questionnaireThe completion of the questionnaires was at the discretion of the participating parents/patients.<br>Questionnaires were filled out by pen and paper and afterwards transferred to a database (Microsoft Access). Double data entry from two independent persons was used to prevent entry errors. Before analyses, a third person reviews and corrects discrepancies in both databases.<br><br>For antibody detection, the serum samples were examined at a 1:101 dilution using a commercial anti-SARS-CoV-2 S1 IgG ELISA kit according to the manufacturer’s instructions (EUROIMMUN Medizinische Labordiagnostika AG, Lübeck, Germany).
Timing	Data collection started at 1 Jun 2020 and ended at 31 Oct 2021. This analysis included all data collected from June 2020 to May 2021.
Data exclusions	All data were excluded where no questionnaire was available (n=1034) or the blood sample could not be analysed (n=63). In addition, data were excluded with missing date of sample collection (n=82) or missing information on the participant's age (n=95). Participants with other missing information in the questionnaire were not excluded from the analysis.
Non-participation	We have no information on how many people declined the study participation or for what reasons.
Randomization	Randomization was not relevant as this is an observational study.

Reporting for specific materials, systems and methods

We require information from authors about some types of materials, experimental systems and methods used in many studies. Here, indicate whether each material, system or method listed is relevant to your study. If you are not sure if a list item applies to your research, read the appropriate section before selecting a response.

Materials & experimental systems

n/a	Involved in the study	
☐	☒	Antibodies
☒	☐	Eukaryotic cell lines
☒	☐	Palaeontology and archaeology
☒	☐	Animals and other organisms
☐	☒	Human research participants
☒	☐	Clinical data
☒	☐	Dual use research of concern

Methods

n/a	Involved in the study	
☒	☐	ChIP-seq
☒	☐	Flow cytometry
☒	☐	MRI-based neuroimaging

Antibodies

Antibodies used

We used a commercially available anti-SARS-CoV-2 Enzyme-Linked Immunosorbent Assay (ELISA - Euroimmun Medizinische Diagnostika AG, Lübeck, Germany) to detect IgG specific for the S1 domain of SARS-CoV-2 spike protein.

Validation

Serum samples were sanalysed at a 1:101 dilution using the automated EUROLabWorkstation ELISA platform. The ELISA yields an optical density (OD) ratio, the quotient of OD in a sample and OD of a calibrator tested in parallel, providing a semi-quantitative measure for antibodies in serum sample. We considered samples with an OD ratio above 1.1 as Anti-SARS-CoV-2 IgG positive, as recommended by the manufracturer.
Method was described previously: Jahrsdörfer, B. et al. Independent Side-by-Side Validation and Comparison of 4 Serological Platforms for SARS-CoV-2 Antibody Testing. The Journal of infectious diseases 223, 796–801; 10.1093/infdis/jiaa656 (2021).

Human research participants

Policy information about studies involving human research participants

Population characteristics

See above

Recruitment

The study's recruitment of participants took place in 14 different pediatric hospitals in Germany. The recruitment at the hospital level was by patient accessibility and compliance. In case of blood sampling for other clinical procedures (irrespective of the medical purpose), guardians/parents of the pediatric patients were asked to consent to use the blood samples for additional antibody testing against SARS-CoV-2. Children with a corrected gestational age of less than 37 completed weeks, severe congenital or acquired immune deficiencies, immunosuppression due to chemotherapy or stem cell transplantation, treatment due to life-threatening emergencies, and children vaccinated against SARS-CoV-2 were excluded from participation.

Recruiting procedure is unlikely to be related to the serological test result but might limit external validity.
Selection bias in either direction might occur depending on available previous test results of a SARS-CoV-2 infection. We tried to limit this bias by not feedbacking the serological test results to the participants. External validity, however, is difficult to validate since participation was not compulsory and catchment areas of the respective hospitals are not clearly defined.

Ethics oversight

Initial ethical approval was obtained from the Ethics Committee of the Medical Faculty of the Heidelberg University (No. 2020-536N). Ethics committees of each all other study centre subsequently also independently approved the study protocol (Ethics ommittee of the Medical Faculty of the Ludwig-Maximilians-University Munich No. 20-348, Charité Berlin, Technical University Dresden No. BO-EK142042020, Medical Faculty of the HHU Düsseldorf No. 2020-936, Saarland Medical Association No. 65/20, Hamburg Medical Association No. MC-142/20, Nordrhein Medical Association No. 2020099, Albert-Ludwigs-University freibug No. 243/20, Medical Faculty of the RWTH Aachen No. 081/20, Medical Faculty of the Justus-Liebig University Giessen No. 61/20, MMH Hannover No. 9041_BO_K_2020, Julius-Maximilians University Würzburg No. 92/20_z, Technical University Munich No. 264/20 S).

Note that full information on the approval of the study protocol must also be provided in the manuscript.
